# Prospects, achievements, challenges and opportunities for scaling-up malaria chemoprevention in pregnancy in Tanzania: the perspective of national level officers

**DOI:** 10.1186/1475-2875-7-135

**Published:** 2008-07-22

**Authors:** Godfrey M Mubyazi, Ib C Bygbjerg, Pascal Magnussen, Øystein Olsen, Jens Byskov, Kristian S Hansen, Paul Bloch

**Affiliations:** 1 National Institute for Medical Research, Dar-es-Salaam, Tanzania; 2 Amani Medical Research Centre, Muheza, Tanzania; 3 University of Copenhagen, Institute of International Health, Immunology and Microbiology, Faculty of Health Sciences, Denmark; 4 University of Copenhagen, DBL – Centre for Health Research and Development, Faculty of Life Sciences, Denmark; 5 University of Aarhus, Institute of Public Health, Department of Health Services Research, Aarhus, Denmark

## Abstract

**Objectives:**

To describe the prospects, achievements, challenges and opportunities for implementing intermittent preventive treatment for malaria in pregnancy (IPTp) in Tanzania in light of national antenatal care (ANC) guidelines and ability of service providers to comply with them.

**Methods:**

In-depth interviews were made with national level malaria control officers in 2006 and 2007. Data was analysed manually using a qualitative content analysis approach.

**Results:**

IPTp has been under implementation countrywide since 2001 and the 2005 evaluation report showed increased coverage of women taking two doses of IPTp from 29% to 65% between 2001 and 2007. This achievement was acknowledged, however, several challenges were noted including (i) the national antenatal care (ANC) guidelines emphasizing two IPTp doses during a woman's pregnancy, while other agencies operating at district level were recommending three doses, this confuses frontline health workers (HWs); (ii) focused ANC guidelines have been revised, but printing and distribution to districts has often been delayed; (iii) reports from district management teams demonstrate constraints related to women's late booking, understaffing, inadequate skills of most HWs and their poor motivation. Other problems were unreliable supply of free SP at private clinics, clean and safe water shortage at many government ANC clinics limiting direct observation treatment and occasionally pregnant women asked to pay for ANC services. Finally, supervision of peripheral health facilities has been inadequate and national guidelines on district budgeting for health services have been inflexible. IPTp coverage is generally low partly because IPTp is not systematically enforced like programmes on immunization, tuberculosis, leprosy and other infectious diseases. Necessary concerted efforts towards fostering uptake and coverage of two IPTp doses were emphasized by the national level officers, who called for further action including operational health systems research to understand challenges and suggest ways forward for effective implementation and high coverage of IPTp.

**Conclusion:**

The benefit of IPTp is appreciated by national level officers who are encouraged by trends in the coverage of IPTp doses. However, their appeal for concerted efforts towards IPTp scaling-up through rectifying the systemic constraints and operational research is important and supported by suggestions by other authors.

## Background

The consequences of malaria effects in pregnant women are widely documented, including anaemia, inter-uterine growth retardation, illness episodes, low birth-weight, pregnancy related complications including stillbirths, prenatal, postnatal or neonatal deaths and other consequences [1]. Malaria is mostly concentrated in sub-Sahara Africa (SSA) where pregnant women and young children under five years of age are the most vulnerable [1,2]. Protecting pregnant women and underfives through use of safe and effective malaria preventive and treatment methods is recommended [3], including intermittent preventive treatment (IPT) and insecticide-treated nets (ITNs), among other methods [2,4]. The World Health Organization (WHO) recommends at least two doses of sulphadoxine-pyrimethamine (SP) to be administered by health workers to women attending antenatal care (ANC) clinics for IPT during pregnancy (IPTp) [5]. The guidelines for IPTp administration insist direct observation (DOT) of pregnant women taking SP under the IPTp strategy [5-7]. Sadly, IPTp coverage is still low in most of SSA countries especially for the second dose [8-10], as most women get the first dose [11]. Current research and policy debate focuses on the operational effectiveness and coverage of IPTp. This includes the actual and perceived benefits, cost and risks of IPTp-SP as well as the feasibility of IPT in the health care system, where other interventions such as ITNs are implemented concurrently [10,12]. Reproductive and child health (RCH) services in SSA as in other developing countries involve both the government and non-government sectors and the role of the latter sector is more recognized now than before. However, little is known about the performance of that sector and its interaction with the public sector in RCH including ANC [13].

Tanzania has the third largest population at risk of malaria [14] and malaria is the leading cause of morbidity and mortality of about 35 million people in the country. The malaria control budget consumes 39% of the total health expenditure [15]. In about 1.7 million pregnant women facing malaria each year, 20% die of the disease, which contributes to 1.3% reduction in the national economic growth [16]. IPTp is implemented throughout the country as part of an essential ANC package [17-19]. Until 2003, Tanzania indicated a remarkably high ANC coverage of about 80% of pregnant women completing at least two ANC clinic visits [5]. Yet, many women are facing problems in accessing appropriate obstetric care and are facing pregnancy complications attributable to untimely access to maternal health services [20-24]. As reported from other SSA countries [11], the government recommends the delivery of RCH services to the vulnerable groups free of charge, and this has been of the key essential elements of the national health sector reform policy strategies in Tanzania [25,26]. Officially, IPTp with SP was recommended in 2001, but became effectively implemented countrywide several months later after orientation/training of frontline health workers [17]. IPTp is concurrently implemented with the national discounted voucher scheme whereby the pregnant women attending ANC clinics receive a special voucher for them to access ITNs at subsidized prices [27,28]. However, lessons have shown that not all the pregnant women from poorest households have access to nets at the subsidized prices [29]. There are concerns about the possibility of unavailability of the ITNs at ANC clinic level and inability to redeem the ITN vouchers negatively affecting women's ANC attendance behaviour and access of IPTp services (Dr. Mufungo Marero – National Malaria Control Programme (NMCP), Dar-es-Salaam, per comm.). This concern is supported by evidence from Mkuranga and Mufindi districts. Public and private HFs participate in the provision of RCH services including ANC, but the presence of user fees for ANC dissuade pregnant women's utilization of some of the HFs [30]. Inaccessibility of HFs providing ANC especially in remote areas where even outreach services are difficult to be carried out is an impediment to pregnant women's utilisation of ANC services, hence raising questions about the possibility of reaching high IPTp coverage [31].

## Objective of this paper

The information presented in this paper is part of a larger study aimed at exploring the economic and other contextual determinants of the acceptability and practicability of IPTp in different district settings in Tanzania [32]. The paper presents the views of the officers working at the Ministry of Health and Social Welfare (MOHSW) and those at the NMCP headquarters regarding the achievements, challenges and opportunities for IPTp in Tanzanian districts that are implementing IPTp within the ANC service settings.

## Methodology

### Study design

This is a descriptive part of a broad study that was cross-sectional and exploratory in nature. It was aimed at collecting specific policy, planning and management information from key persons involved in policy, planning and management aspects for malaria control at national level and from two case study districts (Mkuranga and Mufindi) implementing malaria IPTp guidelines. The design of the research instruments used in the two case study districts took into account of the suggestions from some of the national level malaria control programme officers who were consulted to obtain their views on operational issues which required research. The officers were also informed that they would be approached for interviews to supplement the information that might be provided by the district level respondents. Further information used in the study design process was based by studies previously undertaken in four Tanzanian districts, namely, Korogwe and Muheza [17,33] with stable malaria, and Temeke and Ilala districts in urban Dar-es-Salaam, with stable malaria. These studies primarily looked at psychosocial and systemic determinants of acceptability and practicability of IPTp from the viewpoint of stakeholders in particular the ANC workers, ANC users and district council health management teams (CHMTs).

### Study population and their sampling criteria

The selection of the study participants in this sub-study was *purposive*, as it accounted for their positions and experiences in line with the main study objectives and study questions. Table 1 summarizes the details of the officers approached and actually involved in the study. It was not possible to trace the representatives of the private-for-profit health service providers, but views were obtained from the representative of the private-not-for-profit (faith-based) organizations, particularly the Christian Social Services Commission (CSSC) of Tanzania (Table 1). Generalizability of the findings from small sample in qualitative research is possible as long as the researchers comply with the standards of selecting the cases carefully and explicitly to ensure triangulation of the data from different sources that are representative of the absolute or closely real situation [34,35].

**Table 1:** Officers approached for interview on malaria IPTp operational issues at national level

Respondents	Selection criteria and sample size
*NMCP officers*	Five officers at the national malaria control programme (NMCP) were involved, including the Manager and Deputy Manager of the NMCP, two officers from the Case Management of Malaria (CMM) and Malaria in Pregnancy (MIP) Unit and the Epidemiology Unit. Officially, these officers: (a) are responsible for planning, supervision, monitoring and evaluating of NMCP activities in all the 121 districts in Mainland Tanzania; (b) regularly interact with malaria policy-makers and private sector partners on malaria control issues and do receive reports from all the districts which they use for execution and developing national strategic plans for malaria control (Mwisongo et al., 2007 [49]); (c) periodically supervise, monitor and evaluate the implementation of district health plans (Dr. Alex Mwita, other NMCP officers per comm.).
*MOHSW Officers*	Two officers including the former Director for Preventive Services (DPS) and one Pharmacist from the Pharmaceutical Unit were involved. The officer who was acting as the DPS from late 2006 was approached for at least personal communication. The former DPS who retired in 2006 provided detailed information based on his experience with interaction with the NMCP and MOHSW on consultancy activities. His successor was appointed by the President in 2007 while this study was in the last stages of its implementation. As a former regional medial officer, he had was considered for interacting with the national level officers on NMCP issues.
*Representative of Private health-care service providers*	The private sector agencies involved in health care service provision was represented by the Faith-based (Church) organizations' representative, particularly the Christian Social Services Commission (CSSC) of Tanzania.

*NMCP – National Malaria Control Programme

### Data collection methods

The information was collected through conversation kind of face-to-face in-depth interviews with the officers concerned, when they could find a convenient time. As agreed with the officers concerned when the study was introduced, telephone interviews were also undertaken when the officers could not be physically met at times they were faced with work-pressure. Two officers, including the Acting DPS and the NMCP Manager could not secure time for in-depth interview, but they contributed their views in brief through personal communications when they were approached. They also suggested that the information provided by other interviewees at the NMCP would provide a sufficient insight and the viewpoint of the NMCP. The face-to-face and telephone interviews were conducted several times between November 2005 and October 2007. This was aimed at obtaining updated information concerning the status of IPTp implementation and coverage in Tanzania based on the progress reports from national surveys that would be available at national level. The study team was informed that the national first-line antimalarial drug for uncomplicated malaria SP would be officially replaced by an artemisinin-based drug combination therapy (ACT) any time within year 2006 while the present study would still being implemented [33,36], however, ACT would not be used for pregnant women. This allowed follow-up data to be collected in relation to IPTp acceptability after official implementation of the new first-line drug policy. Interviews were not record-taped, but the key points were carefully taken by hand and updated as the interviews took place several times. This allowed additional details to be taken to fill the gaps in the notes taken previously, including necessary editing of the notes. An interview guide with a list of the study themes was used. Broadly, these issues covered were related to: (*i*) the national IPTp coverage and factors for the recorded coverage levels; (ii) national guidelines for IPTp service delivery in terms of their relevance, recommended number and timing of the doses, financing mechanisms and practicability, including district CHMT financial plans and actual resource allocation for the ANC services; (*iii*) private-public-partnership in ANC services; (iv) acceptability of SP for IPTp after SP has been replaced with ACT, particularly artemither-lumefantrine (ALu) (or coartem) as the first line drug for the treatment of uncomplicated malaria; (v) role of the media in promoting malaria in pregnancy control strategies including IPTp; (vi) health management information system role in terms of records related to IPTp coverage; as well as the (*v*) contemporary challenges, measures in place to face such challenges, and opportunities for scaling up IPTp services towards meeting the predetermined policy goals for the prevention of malaria in pregnancy through ANC services. Several documents cited as references by the respondents in support of their arguments, follow up was made by the principal investigator for confirmation and appropriate citation for the study.

### Data Process and analysis

The brief field notes were expanded in the notebooks by hand and then using a word processor. Transcription of the information collected was done immediately after data collection, but were updated information from additional interview sessions [35]. Data were analysed manually using a qualitative content analysis [34,35] according to the pre-defined study themes. The data were categorized thematically by identifying and discussing the points or explanations that seemed similar or different in relation to a particular study issue. After transcription of each individual interview case, the individual respondents were asked to comment on the transcriptions of results presented in the manuscript, and any new/additional ideas were considered accordingly. This was done so as to validate the data by ensuring that the investigators have not imposed their own constructs that were contrary to the perspectives of the respondents.

### Ethical considerations

The study obtained ethical clearance from the MOHSW through the Medical Research Coordinating Committee. The respondents were asked for and agreed to participate in the study. They were assured confidentiality and their freedom of their decision to participate or withdraw from the interviews as they wished without consequences. No financial incentives were given or promised to the respondents and the officers accepted the findings from their interviews to be published.

## Results

### IPTp coverage in Tanzania

Tanzania was represented in signing the Abuja Declaration in 2000 which set a target of 60% coverage of pregnant women with IPTp and ITNs by end of 2005 [18], although the attainment of that target would depend on the accessibility of financing from the Global Fund by the NMCP and its partners, cooperation and coordination at national and district levels. Given the records on remarkably high national average ANC attendance rate of 80%–98% [5,10,37], Tanzania went further by extending the target for IPTp coverage up to 80% by 2008 and with prospects for additional funding and commitment at national level, this was considered attainable.

**Figure 1:**
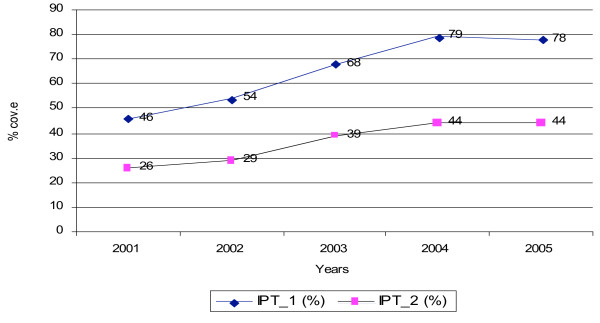
Shows the trends in the coverage of the doses of IPT for malaria during pregnancy in Tanzania between the years 2001 and 2005 based on the national survey of 21 districts.

However, the IPTp coverage especially the second dose (popular as IPT2) is still low despite variations between districts. Contradictory statistics were given regarding coverage of IPTp doses, indicating a weakness in the health information recording and reporting system. Until March 2007, a survey report available at national level (particularly at the NMCP level) showed data collected from 21 districts (1 district per region) indicating IPT1 and IPT2 coverage of about 65% and 45%, respectively [38]. The latest report of the NMCP [19] indicated IPTp coverage for IPTp service to have increased from 46% for IPT1 and 26% for IPT2 in 2001 to 78% and 44% for IPT1 and IPT2, respectively in 2005 (Figure 1).

This trend encouraged only three NMCP officers. The rest expressed doubts about the (i) attaining the new 80% target of IPTp coverage by 2008 due to various constraints as explained in the subsequent sections (ii) unreliability of the IPTp coverage data including data on actual IPTp doses administered at HF levels (iii) reports from several districts indicting ANC attendance rate of 54.4% [38]. As argued, the IPTp coverage data for 2006 and 2007 available at the NMCP were extrapolated from the evaluation report of 2005. In this evaluation, one district from each of the 21 regions was evaluated. The use of extrapolated data does not reflect the real coverage on the ground and therefore there is need for systematic research. One officer showed data on the estimates of IPTp coverage for 2006, whereby it was noted that IPT1 and IPT2 was 62.0% and 41.6% respectively, which are decreasing rates compared to year 2005 records. This is because in 2006 there happened stock-out of SP in a number of districts in the country as records indicate [7] and as reported from district CHMT. Moreover, the NMCP in the last two years failed to undertake monitoring and evaluation activity on IPTp and ITN services in all regions and all the planned supervision due to shortage of funds from donors.

### ANC and IPTp services by private and public health providers

All the respondents commended the government for emphasising private and public partnership (PPP) in health service provision. This was in recognition of the role of the private sector in health services. Non-government organizations (NGOs) have formed the Tanzania NGO Alliance Against Malaria [18]. There is also an Association of Private HFs in Tanzania (APHFTA) that is a partner to the RCH Unit of the MOHSW. Through APHFTA, issues related to private sector providers' compliance with the national guidelines are emphasized and this was viewed as an opportunity for strengthening malaria interventions. The distribution of private and public HFs was considered to have an impact on the coverage of IPTp services. General views indicated that in most rural areas ANC services are provided at government dispensaries, as the few private (especially the for-profit) ANC clinics available are concentrated in towns. In areas like Kagera and Dar-es-Salaam regions, where the private HFs either dominate or are highly utilized (especially the faith-based HFs) in a number of areas, several respondents doubted about compliance with IPTp delivery guidelines and coverage of IPTp doses. Therefore, the importance of assessing the performance of the non-government sector agencies and the challenges they face in malaria control programmes was acknowledged. Overall, government HFs provide 80% of ANC services and the private sector contributes 20% out of which 11% are delivered at faith-based clinics [39].

The issue of adherence to national ANC guidelines at private and government HFs was considered a big challenge. The respondents could identify the causes of this non-adherence situation, but seemed to have no clear-cut answers as regard to how this challenge can be overcome. Four officers (including the representative of the CSSC) argued that this challenge existed because the support to private sector providers with necessary supplies and training was inadequate.

### Determinants of ANC utilization and IPTp coverage

All the officers interviewed considered the most important factors contributing to low coverage of IPTp doses in Tanzania to include the following:

#### Late ANC booking

**Figure 2:**
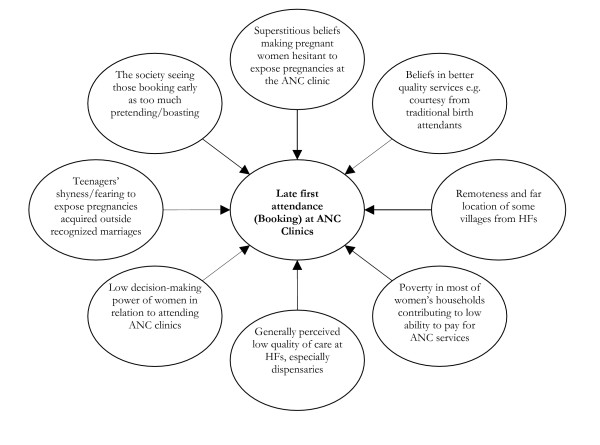
Shows a summary of the factors that contribute to late booking for ANC by the pregnant women in Tanzanian districts.

Late ANC attendance (that is, *visiting ANC clinic in the period outside the recommended gestational age or weeks of pregnancy for receiving the basic health care services*) by a considerable proportion of pregnant women especially in rural district settings was reported to contribute to low coverage of IPTp doses. It was noted that the late attendees ultimately receive only one dose of IPTp or none. The respondents testified to have been receiving reports from district CHMTs indicating various economic, psychosocial and cultural barriers to pregnant women registering early for ANC (Figure 2).

**'*Lack of human touch*' **by the ANC workers is one of the elements of the perceived poor quality of ANC especially at dispensary levels, discouraging pregnant women to book early or regularly contact clinics. As noted above, late booking was defined as the time of booking when the pregnancy is aged 20 weeks or more. As noted in one study, 76% of the ANC workers interviewed in 12 districts showed poor interpersonal communication skills with their clients [7]. None of the officers viewed that the late ANC booking and irregular attendances problems could be resolved by the health authorities/sector alone. One officer warned that the women who book late should not be blamed as most of these women face socio-cultural and other access barriers for attending clinic (Figure 2).

#### User charges for ANC care

Controversial views were obtained regarding whether or not user-fees/charges for ANC services (including IPTp) were implemented at public and private HFs. Three respondents from the government side were confident of their experience with user-charges at non-government HFs. The rest did not believe so, but could also not deny such a possibility. Several respondents viewed that it was inevitable for the private providers to impose the charges. Otherwise such providers could not recover their operational costs, particularly in situations where the district CHMT does not supply free SP. Four officers testified that private clinics charged the ANC clients for laboratory services and some drugs other than SP, hence disappointing the poorest to contact such providers. The point of un-affordability of user-charges at private facilities was contradicted by two other officers who viewed that the actual rates were too low to discourage ANC attendances. Mistrust about private providers delivery of IPTp for free was expressed further by the Deputy NMCP Manager, former DPS and two other officers suggesting the need for researchers to assist the NMCP in evaluating the private agencies' adherence to the national ANC guidelines of free delivery of IPTp among other ANC services and how their practices might have affected ANC attendances and coverage of IPTp. However, the existence of user charges for ANC services in general including SP delivered for IPTp at non-government facilities was confirmed by the NMCP Manager and the Representative of the CSSC. The latter officer claimed:

"*There is no compensation for exemptions from the government, so how can the private service providers afford continuing to provide free services without user fees? Even at the government facilities not all the eligible people are exempted as the policy recommends. Last year (2006), Church agencies submitted their requests for compensations but up to now (October 2007) they have received nothing. The public has to bear with us, I am afraid".*

Accordingly, a survey of 71 HFs undertaken in different regions found that 22 (23%) of the HF were charging patients for IPTp [7].

Moreover, the CSSC Representative criticised the government providing contradictory policy directives while knowing the impracticability of such directives. It was argued:

"Since the introduction of a new first-line drug against uncomplicated malaria in 2007, the government has not provided subsidy to private providers while insisting the providers implementing fees to charge less than 500 shillings per dose. In the market one dose costs 10,000–12,000 shillings. How can private providers afford? I wonder because pregnant women are noted of failing to pay 300 shillings per a dose of SP"

While the majority of the government officers believed that non-adherence with delivery of free ANC services was most likely to be in the private sectors' side, the head of the CMM and IPTp Unit cautioned contrary to this point and in support of the former:

"We need to be realistic not to point a finger to the private providers who charge the women for IPTp if they are not supplied with SP and yet are not compensated anyhow for the cost they would incur by providing SP free of charge".

The NMCP Manager viewed the difficulty of enforcing the private sector to comply with IPTp guidelines due to lack of an official government regulatory mechanism. He emphasized that the exemption policy for RCH services is meant for the government HFs only and people who contact private providers do so at their discretion while being aware of the existence of user-charges and their eligibility for free services at government HFs. Referring to a recent evaluation by the Ministry of Health and Social Welfare (MOHSW) in collaboration with other agencies [13], one officer reported the existence of unofficial charges for RCH services at government facilities contrary to the government policy [20,25,26]. This point was, however, not believed by the rest of the officers.

#### Supply of SP

HWs' adherence to the administration of IPTp doses was linked to depend on the availability of SP at HF levels, besides clean and safe water and skills and staff workload. CHMTs were considered responsible for distributing drugs for IPTp to all HFs delivering ANC services regardless who owns such facilities, in line with expectations for the Expanded Programme for Immunization (EPI) under which the vaccines are distributed to all of the government and non-government facilities.

Two officers from the MOHSW were uncertain about the private ANC clinics receiving SP supplies from CHMTs. It was confirmed by other two officers from the MOHSW that the private-for-profit ANC clinics were not supplied with SP to avoid pregnant women paying for drugs they were supported obtain for free. Others reported the failure of CHMTs to support the private HFs regularly in fear of such providers possibly charging their clients irrespective of the drug supply supported from the government without cost. The former DPS suggested that private sector providers should not feel convicted for charging their clients contrary to government guidelines and are not marginalized since they sometimes fail to respond in time to the calls from the MOHSW asking them to submit their priorities.

The Christian Social Services Commission (CSSC) correspondent revealed that on the 1^st ^October 2007, an evaluation report by the CSSC in Mwanza region indicated that the church owned HFs in that region had not been administering IPTp-SP due to lack of subventions for or supply of SP from the government. The NMCP and three other officers reported the experience with temporary shortage of SP even at government HFs in the country in 2006 due to logistical constraints that made the Medical Stores Department (MSD) at central level to distribute the drug kits. This resulted in pregnant women eligible for IPTp not accessing SP.

District CHMT concern about experience with drug budget shortages was expressed, and this was generally viewed as a weakness as they had never received negative response from the MOHSW to direct the MSD to act accordingly. However, it was not elaborated whether a maximum response time had been defined and adhered to. Some national officers reported the failure of district CHMTs to submit requests of extra drug kits to the central government that despite the government having already set fixed budget ceilings for each district, consideration could be made at central level. This was not the case due to negligence of the responsible district officers to monitor drug stock-outs at HF levels based on which the districts could submit their requests in time. One officer expressed sympathy to staff working at private HFs in remote areas who due to stock-out of essential drugs (including SP) at their HFs are forced to travel in follow up of support at the DMO's office in the district capital. Yet such workers end up incurring expenses on travel, food and sometimes accommodation (when they spend a night in town) while being uncertain of getting compensation (refund) for the costs they have incurred. Such providers occasionally delay or completely fail to submit various reports to the districts due to transport related problems. This point was opposing the point given by the MOHSW's pharmacist that the private ANC providers not always respond in time when they are called to submit their requests for drugs in time to be included by the district health plans that are submitted by CHMTs to the MOHSW. Another officer added that the CHMTs should not be accused of failing to supply SP to private HFs in the absence of instructions from the central level. However, no explanation was given by this officer concerning why the centre has remained silent on this issue.

#### Effectiveness of the Direct Observed Therapy (DOT) procedure

The respondents acknowledged DOT being more effective currently than before, albeit this varying by levels of HFs and locations. Three officers viewed the problem of water shortage being bigger in urban HFs where ANC attendances were very high on some days. In rural areas, water supply for IPTp was considered as being available and adequate. This opinion was contrary to experience given by the frontline HWs and district CHMT in the study districts where occasionally the clients were allowed to take SP at home due to water shortage. From the WHO-Country Office Report obtained at the NMCP it was noted that in 12 districts DOT was impracticable at 7% of the rural HFs and about 2% of the urban HFs [7].

One officer questioned about ANC workers not emphasizing ANC clients to adopt the same system as the tuberculosis (TB) patients, who carry water for taking anti-TB drugs under DOT. Nonetheless, the officers acknowledged that that understaffing at a number of HFs contributed to occasional failure of the DOT to be implemented even if water was adequately available. Simultaneously, suggestion was given that the shortage of water could be reduced by the district council authorities prioritizing budget for water in their annual health budgets. According to Drs Sigsbert Mkude and Mufungo Marero at NMCP, the financial constraints faced by the districts might not allow the budget for water to be considered in the district health plans while other two officers argued that some districts were financially able but neglecting to prioritize the budget for water.

#### ANC workers' skills in administering IPTp

Low skills of ANC workers in complying with the national guidelines were considered another constraint to IPTp delivery and coverage. Some HWs failing to measure the fundal (uterus) height contributes to eligible women not receiving IPTp at the right time, it was reported. As noted by three officers, the latter explanation criticises the general preconception by health professionals that late-first attendance is the main cause of pregnant women's low uptake of IPTp doses. It was testified:

*"I have noticed HWs somewhere sending back home the pregnant women with a two months pregnancy on ground that it was too early for them to book. These women must be discouraged and might not find it useful to revisit the clinic immediately" *(former DPS).

The nurse auxiliaries (medical attendants) were reported as being the most inadequately skilled staff in identifying women's pregnancy conditions and understanding the national focused ANC guidelines. A continuous use of such unskilled nursing personnel was associated with the general shortage of the skilled nurses (that is, midwife and public health nurses). The owners of HFs are advised to employ qualified staff and provide on-job training to upgrade their skills, but in practice, private HFs face a bigger shortage of skilled staff as the employers opt for cheap (but unskilled) labour. The former DPS cited Kenya where the training of HWs demonstrated a remarkable improvement in IPTp implementation and coverage [40], but for Tanzania there is yet inadequate systematic evidence on this issue despite acknowledgeable arrangements made to orient HWs.

#### Recording system for IPTp doses

HWs' low skills and understaffing contributing to poor recording of the IPTp doses delivered at HF level was reported by all the respondents to raise concern about the real coverage of IPTp. All of the officers discussed the work-pressure facing the inadequate number of HWs especially at periphery dispensaries. One officer remarked that most of the health statistics taken from HFs are considered by the low level HF staff as being only useful to the district CHMTs and MOHSW. Such workers especially in remote areas where sometimes the CHMT does not strictly reach for supervision see no immediate advantage of documenting systematically the IPTp doses as they do for the services related to TB, HIV, notifiable diseases and vaccination services usually monitored by their programme officers on weekly or monthly basis. As an example, acknowledgement was made about the EPI under which strict supervision of HFs on EPI services. This includes the keeping of records because the EPI is treated as a sensitive issue from global and national programme levels through to CHMT level. Moreover, the design of the current health management information system (HMIS) registers (popular as *MTUHA in Tanzania*) was noted of weaknesses, giving the frontline HWs difficulty in keeping IPTp records.

#### ANC workers' knowledge on SP doses

CHMT and frontline ANC workers in Mkuranga and Mufindi districts reported confusion about the timing of IPT1 and IPT2 and the recommended number of doses. Their concern was considered by the national level officers, however, in a controversial manner. The head of the CMM and MIP Unit thought that poor administration of IPTp doses is due to some HWs being lowly knowledgeable on IPTp guidelines as they have limited opportunities for training. HWs administering IPTp doses in the period outside the recommended weeks of pregnancy was linked to weaknesses in the national focused ANC guidelines which emphasize the administration of IPT1 during the 20^th ^week and IPT2 during the 32^nd ^week.

ANC guidelines have been revised, but the revised version has not been circulated to districts because they had not been printed, it was revealed. The initial guidelines specify that SP can be given after the 16^th ^week for the pregnant women who book early and attend regularly. This means, if the staff administered SP between the 17^th ^and 19^th ^week, they were still right as long as they adhered to the interval of four weeks between one dose and another. It was remarked by the head of the CMM and MIP Unit:

"The problem I can see is where the revised guidelines still give bold highlight on the 20–24^th ^weeks for the first dose because experience has shown that most of the pregnant women do not attend ANC clinic before 20 weeks of pregnancy".

Based on experience from previous supervisions in several districts, the latter respondent revealed tendency by some ANC workers to administer IPT1 only in the 20^th ^week or 24^th ^week and giving IPT2 during the 28^th ^or 30^th ^or 32^nd ^week and skipping any other week falling between the stated intervals of 20–24^th ^week and 28^th^-32^nd ^week.

Conversely, another officer expressed great confidence in the current ANC guidelines, by claiming:

"Some of those who claim to have heard differently from their colleagues who attended seminars have a hidden agenda like wanting to be invited to seminars where they could also receive some allowances that are normally paid to the participants".

Doubt about the confusing directives related to administration of IPTp doses was shown by two other officers. These respondents appealed to the HWs attending various workshops/seminars to avoid unofficial views of the seminar organisers that are contrary to the specifications of the national guidelines. Regarding the appropriateness of administering two or more doses of IPTp to a woman throughout her pregnancy, reference was made to WHO's emphasis that there is no evidence of additional benefit of administering three or more doses of SP for IPTp to people who are HIV sero-negative.

#### Role of the price-subsidized insecticide-treated net vouchers on women's ANC attendances

The NMCP Manager, previous DPS and several other officers argued that the voucher scheme has fostered early booking. This point was partly challenged by the head of the CMM and MIP Unit who was of the opinion that early booking is contributed mainly by the perceived good quality of care and women's sensitiveness to pregnancy safety. This is mostly the case for those who have acquired appropriate health education messages either through the radio, based on their previous attendance to ANC clinics or advice from other people (e.g. spouses, relatives, friends). The officer reported 51 districts that run out of stock of the vouchers to distribute to HFs between May and mid August 2007, by speculating that a number of pregnant women must have been disturbed and disappointed to revisit clinics. In some areas/districts the vouchers were not available at the accredited shops or HFs, though sometimes the ITNs were available but the individual women failed to top up cash to buy the net [10]. Only two officers thought this would hinder some women to attend clinic, particularly those who might expect being mocked by the HWs after showing inability to redeem the nets [26,28]. It was noted that previous evaluation by CARE Tanzania noted that the pregnant women who failed to top up the vouchers were denied the vouchers, and this is a disappointment to the women concerned and might have discouraged some women to attend clinic (ii) their confusion about and negative perception of the user-fee system. Already, the challenge facing pregnant women from poor households in accessing ITNs is already recognized [41-44]. The reported inability of some women to pay for the nets made the MOHSW to specify a uniform topping up price of 500/- shillings (US$0.4) per voucher [26].

One officer expressed doubt about the contention that discounted voucher schemes fosters early booking for ANC. Such a respondent argued that the policy of pregnant women toping-up nets contradicts itself by applying double standard guidelines since the same policy recommends pregnant women to access RCH services free of charge [26]. Otherwise the country will not be able to attain the Abuja target unless it adopts the same strategy like Kenya, Zambia, Malawi and Togo that have reconsidered moving to free mass distribution of ITNs and Eritrea that has since 1995 been providing ITN free of charge [9].

#### District CHMT support to peripheral health workers

The importance of supervision of ANC workers on improving the quality of ANC services, delivery and coverage of IPTp was acknowledged. Yet, irregular supervision by CHMTs in most of the districts was also pinpointed by the peripheral health workers and confirmed by CHMTs in the study districts. Some of the CHMT officers pretend to have undertaken supervision to HFs by signing in the registers and justifying the paid allowance to please the auditors who being non-health professionals could not question about, and confirm the types of health activities. Although four officers acknowledged that currently there is malaria and integrated management of childhood illnesses (IMCI) focal person (FPs) in each of the districts in the country, they were disappointed with the CHMTs that fail to recognize such FPs while the purpose of creating the FPs' position good, as described elsewhere [18,44]. Moreover, the composition of the CHMT membership tending to exclude the district reproductive and child health coordinators (DRCHCo) and malaria-IMCI FPs raised another concern among some of the respondents, including the head of the CMM and MIP Unit and former DPS. The respondents were concerned that the exclusion or temporary inclusion of the latter officers, especially the DRCHCo in the CHMT is a denial of such officers' right for pushing the agenda in the priority setting meetings, including the issue of health service supervision at the advantage of RCH services. The former DPS commented:

"CHMTs should stop treating the DRCHCo and Malaria/IMCI FPs as spare tyres by naming them as co-opted members of the CHMT rather than being permanent members. It is time now these people are recognized if we aim at attaining the millennium development goals number four and five through active involvement of the key stakeholders".

As expressed, ineffective supervision of peripheral HFs regarding the HMIS contributes to the inconsistencies shown in the data kept at lower HF levels. This means, records given by CHMT to the HMIS Unit at the MOHSW's headquarters each quarter of the year concerning ANC and IPTp coverage may not be reliable.

#### Effect of change of the first-line antimalarial treatment policy on IPTp and Role of the Media

All the respondents were still confidence in the efficacy of SP for IPTp. However, they anticipated a considerable proportion of pregnant women disfavouring SP following the replacement of SP with the new national first-line antimalarial drug – ALu for the treatment of uncomplicated malaria. Despite ALu's introduction as the new first line drug, the IPTp guidelines have remained specifying SP as the drug of choice for IPTp. According to the former DPS, it is not known how this may have had effect on IPTp delivery and uptake, considering the experience that policy change poses operational challenges. This is because the government makes policy change decisions before adequate preparations for the change at other levels, including supply of essential drugs at all levels and adequately sensitizing the stakeholders before official announcement:

"We sometimes do a great mistake to give out a press release about the government policy change decision before we are sure that we have adequately prepared for the change. It happened in the past when it was announced that polio has been completely eradicated, causing the vaccine manufacturers to stop vaccine production. Suddenly we faced a situation when we urgently needed vaccines but where to get it!"

Speculative media reports concerning parasite resistance to SP was pointed out to potentially mislead the public. Two officers cited publications on this issue [36,45]. Also, three officers showed one of the local newspapers, namely *Mtanzania *dated 18^th ^February 2007, which was published one day before the day they were interviewed. This newspaper wrote: "*SP is not appropriate for IPTp*". The officers were of the general view that journalists are part of the community and human beings with their own feelings and perceptions on drug policy changes. Therefore, even before they write their stories, they may be biased already or want to make a catchy story that also sell their newspapers. Inadequate use of the media to campaign for IPTp was reported as one of the weaknesses, the cost of advertising being a problem. A good solution could be learnt from social marketing of ITNs through the national, private and religious agencies' radios media investments through which various net manufacturers: here bilateral, multilateral, and private agencies have been collaborating with the NMCP to advertise ITNs; similarly the collaboration with the Tanzanian AIDS Commission to advertise contraceptives and anti-retroviral drugs.

#### ANC-IPTp services in the context of district planning and funding mechanisms

IPTp is implemented along with other types of health services within the existing district health care system. The implementation of most of the planned activities depends on the availability of funds for supporting transport of HWs and supplies from the district capital (CHMT office level) to HFs, among other things. This was noted by three officers who elaborated that normally CHMT present their annual budgets and once approved, receive funds for their health services from the central government on quarterly basis. After the funds have been deposited to the CHMT accounts, the CHMT is informed through the district council executive director. Three officers argued that late reception of information about the funds sent from the central level affected the implementation of the planned ANC and other health service activities. Moreover, it was revealed that although it is known that the funds budgeted have to be sent to districts in instalments, sometimes the money for particular activities arrives in portions rather than in full amount. So, DMOs and their subordinate officers have no way out of prioritizing the little funds available for the most urgent activities and deferring paying some allowances for HWs if the funds are little.

The NMCP Manager argued that financial delays faced many sectors and departments, including the NMCP itself, so it is common and unavoidable. The former DPS argued that although it is true the centre partly delay transferring funds to CHMTs that undoubtedly affect ANC among other services, the CHMTs must accuse themselves for delaying to submit their quarterly and annual financial reports. This is partly due to their failure to keep clear accounts records and end up sending technical reports (e.g. health plans or service delivery activity reports) rather than financial reports to the central level.

Another officer said that each month the MOHSW allocates a flat budget of shillings 500,000 (≈ US$500) and shillings 300,000 (≈ US$300) per health centre and dispensary, respectively, and mainly for essential drug purchases. This is too strict when not considering the differences in the population sizes and dynamics in different districts and health service activities carried out. Budget ceilings predetermined at central level for specific services including those related to RCH problems also give little autonomy for the CHMT to arrive at a more realistic allocation from their own viewpoint.

#### Planned versus realized training of frontline health workers

The NMCP has covered the whole country in the training of the trainers on malaria and syphilis in pregnancy and general focused ANC management issues. This involved health care providers from public and private sectors, the police and military institutions, it was reported. The initial Training of Trainers process began by selecting six health personnel from each district who would cascade the process by training others in their own districts. Nevertheless, two officers anticipated possible failures in the training cascading process as they warned against too much expectation from the cascading training at district level due to the financial constraints facing most of the districts. The officers testified that the previous training experience has shown that most of the districts did not roll forward the training process as agreed. As a result, the training did not reach the peripheral HFs and community grassroot levels, hence how the standard practice for IPTp including the issue of DOT and coverage of two doses of IPTp be expected?, they queried. The report from the WHO-Country office indicated that implementation of MIP strategy in line with FANC guidelines and the capacity of the district health-care delivery system facilitated by cascade training and supervision had not systematically been studied [18]. Furthermore, four officers pinpointed the weakness of the CHMTs which inadequately appreciate the role of the non-government ANC providers by giving them few opportunities for in-service training and favouring the government HWs on grounds of financial shortages to invite many stakeholders while directly showing their preconceived biases against the private sector health providers.

### Respondents' suggestions about measures for enhancing IPTp coverage

#### Supervision, Monitoring and Evaluation

Districts commitment in adhering to the planned supervision is important as part of enforcing the implementation of ANC guidelines in relation to IPTp. This should go simultaneously with supply of SP and essential materials to all the HFs delivering ANC services. Building local health workers' capacity in documenting health management data could be improved through supervision by CHMTs and other agencies at lower levels. Additional and timely funding from the centre is imperative for the districts to accomplish this.

#### Training of frontline HWs

This does not only contribute building the capacity of frontline HWs in service delivery issues, but also enhances their working morale by developing a feeling of being recognized for their need for increasing skills in their careers. Interestingly, one officer seemed greatly convinced about the role of training the frontline HWs, citing an example of Kenya where it has been noted recently that the training HWs had improved IPTp service delivery in Kenya [40]. Commends were given to the current NMCP's strategy for integrating cascade training on ANC-IPTp issues in the pre-service curriculum and in (private and government) secondary schools.

#### Districts' autonomy to budgeting and spending

Cascade TOTs at district level would be facilitated by giving the districts more autonomy to use the budget allocated for health services unlike at present when the training issue is centrally planned. District health plans should clearly justify how the private agencies will be involved and supported out of the budget set for health service priorities. They should also be periodically evaluated and accountable for their plans. Improving the planning skills at district level is urgent to enable the districts utilise the funds for health service activities more efficiently.

#### Supply of essential drugs, ITN vouchers and other materials

A successful delivery of IPTp services requires the reliable supply of essential drugs and non-drug materials. Pregnant women do not attend clinic for purposely or only for receiving IPTp, but also for other services including other desirable drugs and materials such as ITN vouchers. Therefore, the authorities need to look at IPTp implementation in the broader context of health services.

#### Improved Information-Communication-Education approaches

This is not a question of distributing brochures and posters to HFs, but concerting efforts to actually educate the mothers and community at large through diverse means such as use of village HWs and traditional birth attendants. This should be emphasized more by involving men and being more open on the issue of reproduction at family and schools (including junior pupils who are entering puberty age) as emphasized in anti-HIV campaigns. This can foster early booking and awareness creation on sensitisation on safe motherhood.

#### Media Involvement

Involving the different kinds of the media (TV, radio and newspapers) in the whole issue of malaria prevention is indispensable. The NMCP in collaboration with the Gates Malaria Partnership through the Centre for Enhancement of Effective Malaria Interventions has since 2002 prioritized media involvement in campaigning for malaria prevention especially using ITNs [44]. This should be sustained, along with the campaigns for IPTp and improving the media reporting about national policy on malaria prevention and treatment.

#### Operational research

The Monitoring and Evaluation undertaken by the NMCP officers cannot and should not be a substitute for systematic research on the coverage and effectiveness of the IPTp services, as commented by the Deputy NMCP Manager and Head of CMM and MIP Unit. The latter officer added that operational research on the impact of the training done to ANC workers in Tanzania in terms of IPTp administration is lacking, yet there is no particular district that has so far demonstrated the role of the training done on improvement of IPTp practicability and malaria in pregnancy situation.

## Discussion

### Achievements of IPTp coverage

The trend of IPTp coverage based on the statistics presented really indicates an improving implementation of the IPTp strategy since it was officially recommended in Tanzania. Although the records on high average national ANC attendance justify the possibility of achieving the national target for IPTp before 2010, as the officers viewed. However the deficiencies in the HIMIS data on coverage collected after taking into account of rural-urban and other geographical differences within and between districts, makes it unclear about the real national average of IPTp. Not everywhere in Tanzania ANC coverage is as high as 60% or above, which means even IPTp coverage is lower in some places [18]. Also, the survey reported showing data on IPTp coverage is inadequate as they represent only 21 districts out of 126 districts for the country as whole. In situations where information is not reliable, particularly depending on the source of information and the skills invested in documenting the data, the ultimate usefulness of the data collected remains limited. In Malawi, one study obtained varying results on IPT coverage among pregnant women. This was after comparing the results from women interviewed at household level about reception of IPTp doses versus those whose ANC cards were observed to confirm the doses given. The coverage rate seemed to be either higher or lower when compared with other source of information – interviews in this case [46].

### Overview of the challenges to the delivery of IPTp doses and adherence to DOT guideline

Although the IPTp strategy is implemented and managed, it is not yet well integrated in the overall service provision including the current information system. The respondents have provided useful information on a number of common operational constraints, although they partly differ in views on the main causes of for this situation. There is a tendency to pinpoint inadequate management, staff skills/competence and other resources at district level and below. Many of these are quite common weakness in the resource poor settings [47] that the NMCP can do little about in isolation. Training HWs is not seen as an absolute solution to the existing IPT service delivery and coverage problem in isolation of other concerted efforts. But, some of the suggested systemic changes in relation to accelerated decentralisation will provide an opportunity for local support to a sustainable increase in coverage provided that it is accepted as a high priority programme in the local setting and resource-constrained situation. Strong health systems are imperative for the success of the planned vertical programs, unfortunately a fragile and fragmented health system fails to allow the delivery of adequate and quality of services to those in need [47,48]. In the meantime the central level may consider exploring available options for supporting NMCP and CHMTs including monitoring jointly with other national special programmes, supporting monitoring, evaluation and relevant operational research.

The view expressed by one officer that the national focused ANC guidelines are clear and straightforward to comply with seems to be negligent of the concern expressed by the frontline HWs who are responsible for translating the guidelines into practice at HF level. This kind of view indicates the attitude of higher authorities/decision-makers who may not value the importance of training of the service providers in improving the service delivery. At least the head of the CMM and MIP Unit at the NMC who felt it needful for training HWs after identifying that the current guidelines overemphasis on two IPTp doses contributes to confuse some HWs. Mwisongo [49] notes that policy decision-making authorities sometimes take it for granted that the guidelines designed at national level are easily implementable. In a situation when there are different agencies giving opposing directives related to drug administration, it is likely that frontline HWs especially those who are semi-skilled (*non-professionals*) would confuse and might end up malpracticing. This is an appeal for such workers not being condemned since studies in Kenya also found HWs confused about IPTp administration, despite the existence of guidelines [11]. Without properly coordinating the intervention activities and agencies on the ground, many chefs playing in the health system may continue giving overlapping directives confusing the already frustrated HWs [50].

The worry expressed by the national officers about low IPTp coverage due to socio-cultural factors hindering ANC attendances is supported by other authors [8,10,11,32]. Moreover, the reported HWs' failure to identify the fundal height, wherefore, contributing to untimely pregnant women's uptake of IPT1 is similar to findings from Malawi and Kenya [11,51]. The issue here is whether it may be a right time for pregnant women to receive the first dose of IPTp when the foetus starts moving, considering that WHO recommends administration of IPTp immediately after quickening [5,52].

The reported concern about shortages of health personnel, drugs and water for IPTp-DOT is validated by evidence from studies documented elsewhere [11,17]. Also, the reported health budget constraints and the bureaucratic situation facing district health authorities partly limiting the performance of the planned health service activities is validated by findings from previous studies [53,54], as well as observed by other authors [48,55,56]. These are systemic operational issues to be confronted.

### User charges for ANC and IPTp

The assumption by some of the present study respondents that the existing rates of user charges for ANC services delivered at private HFs are affordable to all pregnant women is opposed to the findings from previous studies in Tanzania [30,57-59] and other SSA countries [10,60-65]. In Tanzania, most HFs from hospital up to dispensary levels (private and public alike) practice some form of user charges [30,33,39]. However modest the fees might be, the pregnant women get disappointed with paying for ANC cards and screening services at the laboratory and lose trust in the HWs in light of their awareness that the policy recommends them to access the services for free [65].

Concern about costs related to user-charges for RCH services acting as barriers to pregnant women's utilization of IPTp services has been the expressed by other authors [7,8,11,33]. In Kenya some government and private clinics Kenya as were the faith-based clinics in Malawi charged their clients for IPTp and contributed lowering the uptake of IPTp doses [11]. It remains unavoidable for private HFs to charge for IPTp in strive for recovering the operational costs unless there were effective means for compensating them if they granted fee exemptions. For instance, the non-government HFs not being reimbursed for the exemptions granted to their clients made it difficult for them to comply with the government exemption guidelines [53].

### Concurrent implementation of IPTp and ITN strategies

Studies have shown that the delivery of free or subsidized ITNs has potential for foster early booking and coverage of IPTp doses, yet there is limited evidence on this issue [10]. This is in line with the opinions expressed by the present study respondents regarding the challenges to implementing IPTp and ITNs strategies in the same MCH clinic settings. Moreover, it is yet unclear whether the women who are highly sensitized about ITNs and who effectively use the nets find it beneficial to take IPTp-SP doses, especially if they have reservations with SP.

### Media involvement

The media should be part of the policy change and malaria control strategy, as advised by one of the respondents in this study and as emphasized by other authors [45]. It may not sound well to criticise the media announcement or the journalists' articles if such players have not been involved in the process and this includes providing them with the information to print for public use. Quality research can be done but poor dissemination looses the opportunity for targeting the message to the audience to foster better health behaviours and practices. Considering that the media is unnecessarily staffed by health professionals or health researchers who are adequately trained on health reporting, providing the journalists with the written policy messages and research briefs would help to improve communication of health messages [66,67]. Also, the media staff should be sensitised and cautioned on the disadvantage of misreporting, otherwise it sounds meaningless just to invite the journalists at the dissemination workshops/conferences and paying for their publications. Male involvement in RCH matters including sensitizing them about MIP and prevention strategies is essential [8,10]. The media can be an entry point for addressing and targeting the right message to the target recipients, albeit this requires a broader inter-sectoral approach.

### Strengths and limitations of the study

This study presents the views from the key officers at national level who oversee malaria control issues in the country in light of the global advocacy and strategies for malaria control. The similar and different opinions expressed by the respondents reflect not only how malaria chemoprevention strategy is perceived by decision-makers at national level, but also how similar perceptions may influence priority setting for malaria IPTp at lower levels in the health care system. The selection of only two study districts may not reflect countrywide experiences. Nonetheless, previous studies in north-eastern Tanzania [17,33] and a pilot study in urban Dar-es-Salaam reflected a similar situation. Nonetheless (i) most of the respondents were central government employees whose views might not adequately reflected the situation in the non-government sector; (ii) the adoption of telephone interviews might have affected response as the respondents might have hesitated to talk all the important and probably sensitive details through the phones; (iii) the limited time for telephone interviews and the expense incurred on the research institutions and the interviewers by calling the respondents in Tanzania while in Europe; (iv) some useful information might have been lost due to un-tape recording the interviews, and (v) the inadequate sample of the respondents from the private sector. However, this gap has been filled by the information obtained from other government officers about the private sector providers by admitting the gaps identified by the representative of the faith-based providers and acknowledged the role of the private sector in health service provision.

## Conclusion

The benefit of IPTp-SP in improving birth outcomes including mainly the improved body weight of the newborn and prevention of anaemia in pregnancy, premature labour/births and possible deaths to the pregnant woman and/or foetus so far remains acknowledged [2,9,68,69]. All the respondents in the present study appreciated this strategy, however, they were not happy with the low uptake and coverage of IPT2 due to the systemic shortages of skilled HWs, supply of drugs and water (mostly at public HFs) and socio-economic barriers to early and regular attendance for ANC. Truly, these constraints have to be minimized if IPTp services have to be effective and achieve high coverage rate. Systematic training of the frontline HWs, regular use of the media and other means to sensitize the public on ANC services including malaria and IPTp, effective supervision of HFs (both private and public) to monitor service delivery and interfere where necessary, ensuring supply of essential drugs, water, other materials e.g. ANC cards and skilled and adequate human resources at all HFs (private and public), are urgent measures. Moreover, the need for studying the feasibility of new interventions such as IPTp is imperative considering the existence of other interventions such as ITNs, prevention of mother-to-child transmission with for human-immunodeficiency virus and the role of cascade training of frontline HWs and malaria-IMCI FPs on IPTp compliance and scaling-up. We agree with suggestions by other authors that operational research is remains an important integral part of malaria control programmes [12,70-72]. Health systems researchers if financially supported can provide good feedback and suggest alternative ways out by undertaking comparative process and effectiveness studies in the districts with more equipped HFs, human resources and training on IPTp compared with districts or HFs without such kind of support. Scaling up IPTp and other public health interventions really requires concerted efforts, including the translation of the political will in allocating more resources and ensuring effective PPP into a reality. Otherwise each year the story may remain the same as before. Moreover, encouraging pregnant women to attend clinic several times and the administration of IPTp-SP should not be considered to be ultimate effective solution for avoiding anaemia in pregnancy. Taking SP in an empty stomach especially among the women who are poorly fed could be another challenge. Therefore, concerted measures are required to promote improvement in the nutritional status of the target women [73-75], although this remains a challenge in situations of persistent food shortage, as in many developing countries within SSA and elsewhere. Owing to the doubts expressed by one of the respondents in this study, it is crucial for the research scientists (including the general practitioners) to investigate and justify beyond reasonable doubts the possibility of administering IPTp-SP in the gestational age other than the currently recommended by policy guidelines in attempt to foster a higher coverage of the uptake of IPTp by all the eligible ANC clients [71].

## Conflict of interests

The authors declare that the have no competing interests.

## Authors' contributions

GMM conceived the study, participated in all stages of the study as part of his PhD training, drafted the first manuscript (MS) and worked on comments from co-authors. The rest of the co-authors supervised GMM's PhD study and commented on the MS. The supervision was lead and coordinated collaboratively by PB and ICB. All authors read and approved the final MS. The MHSW/NMCP is not responsible to the views expressed by the authors of authors of this paper.
